# Sex-Specific Response to Stress in *Populus*

**DOI:** 10.3389/fpls.2017.01827

**Published:** 2017-10-26

**Authors:** Nataliya V. Melnikova, Elena V. Borkhert, Anastasiya V. Snezhkina, Anna V. Kudryavtseva, Alexey A. Dmitriev

**Affiliations:** Engelhardt Institute of Molecular Biology, Russian Academy of Sciences, Moscow, Russia

**Keywords:** *Populus*, poplar, sex, dioecious species, stress, environment, drought, salinity

## Abstract

*Populus* is an effective model for genetic studies in trees. The genus *Populus* includes dioecious species, and the differences exhibited in males and females have been intensively studied. This review focused on the distinctions between male and female poplar and aspen plants under stress conditions, such as drought, salinity, heavy metals, and nutrient deficiency on morphological, physiological, proteome, and gene expression levels. In most studies, males of *Populus* species were more adaptive to the majority of the stress conditions and showed less damage, better growth, and higher photosynthetic capacity and antioxidant activity than that of the females. However, in two recent studies, no differences in non-reproductive traits were revealed for male and female trees. This discrepancy of the results could be associated with experimental design: different species and genotypes, stress conditions, types of plant materials, sampling sizes. Knowledge of sex-specific differences is crucial for basic and applied research in *Populus* species.

## Introduction

The genus *Populus* includes 29 species as per Eckenwalder’s classification (Eckenwalder, 1996). Most *Populus* species are fast-growing trees, which are distributed in the Northern Hemisphere. *Populus* is one of the best-studied genera among woody plants. The genome of *P*. *trichocarpa* was the first tree species to be sequenced (Tuskan et al., 2006). *Populus* has become a ‘model’ for tree genetic studies because of its small genome size, possibility of genetic transformation, easy vegetative cloning, rapid growth, short pre-reproductive period, and potential for commercial use (Ellis et al., 2010). To date, immense and complicated data on the genetic and epigenetic characteristics of *Populus* are available in the literature, databases, and public resources, such as the plant comparative genomics portals Phytozome^1^ and PopGenIE^2^. *Populus* includes dioecious species (poplars, cottonwoods, and aspens), which have male and female reproductive organs on separate individuals. Recent studies have identified a small (about 100 kb) sex-associated genome region in *P.* *trichocarpa* and *P. balsamifera* (Geraldes et al., 2015). Differences between male and female individuals of *Populus* could not be restricted only to reproduction but also to their sex-specific response to different environments. For their perennial life cycle, *Populus* trees are exposed to a diversity of unfavorable conditions. Stresses, such as drought, salinity, and low temperatures, dramatically affect growth, development, and productivity of trees and play an important role in the geographic distribution of species, including *Populus* spp. (Harfouche et al., 2014). In the present work, we summarized recent studies related to differences between male and female individuals of *Populus* species regarding their response to different stress conditions, including drought, salinity, heavy metals, nutrient deficiency, and elevated CO_2_ concentration.

## Drought

Drought is among the most harmful abiotic stresses that limits plant survival and growth. Differences in the response of male and female *Populus* plants to water deficiency has been examined. Leaf area, total number of leaves, photosynthetic and transpiration rates, and efficiency of photosystem II decreased, whereas total chlorophyll concentration, carbon isotope composition, abscisic acid (ABA) and malondialdehyde contents, and peroxidase activity increased with drought both in male and female plants of *P. cathayana* in chamber and greenhouse conditions. However, female plants were more sensitive to drought environments and exhibited a more pronounced decrease in growth and photosynthetic capacity than that of male plants (Xu et al., 2008a,b). It was suggested that the differences in the response of male and female *P. cathayana* plants to drought were associated with sex-dependent protein expression related to photosynthesis, homeostasis, and stress response (Zhang et al., 2010a). Roots of *P. cathayana* male plants were less sensitive to water deficiency than those of female plants under drought stress. However, the shoots of female plants grew faster than those of male plants under well-watered conditions. Grafting of female shoots onto male roots improved the resistance of plants to drought (Han et al., 2013b). Both the sexes of *P. cathayana* demonstrated a reduction in growth and physiological functions under drought conditions; however, after inoculation with an arbuscular mycorrhizal fungus (AMF), *Rhizophagus intraradices*, male plants showed higher drought protection than female plants (Li et al., 2015a,b). In another *Populus* species, *P. yunnanensis*, female plants experienced more pronounced growth inhibition, reactive oxygen species (ROS) accumulation, and decline in the accumulation of dry matter and total chlorophyll content under water deficiency than that of male plants (Chen L. et al., 2010). The molecular basis of sex-related differences in the response of *P. yunnanensis* to drought stress was determined. Under this stress, alterations in the gene expression levels involved in photosynthesis (photosynthetic electron transport, photosystem I and II, and antenna protein biosynthesis), hormone biosynthesis (ABA biosynthesis), and ROS elimination (genes encoding ascorbate peroxidase, dehydroascorbate reductase and catalase) were more pronounced in males than females (Peng et al., 2012). Thus, it was demonstrated that compared to female plants, male plants of *Populus* species adapt to water deficiency more efficiently, and drought stress inhibits growth, photosynthesis, and ROS protection more strongly in females than in males.

## Salinity

Salinity is a major abiotic stress that suppresses plant growth and development. Cuttings of male *P. cathayana* plants were observed to be less sensitive to salinity than those of females, in which the negative effects on growth and photosynthesis were more pronounced. The Na^+^ and Cl^-^ concentrations in female plants were higher in leaves and stems, but lower in roots than those in the respective organs in males. It was speculated that males have a better capacity to restrict Na^+^ transport from roots to shoots (Chen F. et al., 2010). The effect on female cuttings was more negative than that on the male cuttings and resulted in reduced growth and photosynthetic rates as well as greater Na^+^ accumulation. In males, lower degradation and higher abundance of proteins involved in photosynthesis, hydrogen peroxide scavenging, and stress response was observed (Chen et al., 2011). The growth rate of *P. deltoides* females was higher than that of males in the absence of salinity under intersexual competition. However, under salinity stress, males showed a higher capacity for osmotic adjustment and antioxidant activity than that of females (Li et al., 2016). Higher sensitivity to salinity and a combination of drought and salinity was also observed in *P. yunnanensis* females than in males. The female plants showed a more pronounced reduction in growth rate, higher ROS accumulation, and greater cell organelle damage than that of males (Chen L. et al., 2010). The *P. yunnanensis* female plants were found to be less tolerant to salinity than the males; the female plants exhibited less growth and lower photosynthetic rates than the male plants. Moreover, elevated CO_2_ levels enhanced the negative effects of salinity in females (Li et al., 2013). Transcription profiling revealed that mainly the genes involved in photosynthesis (photosystem II and antenna system) were upregulated in males, but downregulated in *P. yunnanensis* females, under salt stress (Jiang et al., 2012). Additionally, the role of AMF in the sex-associated response of *Populus* to salt stress was investigated. *P. cathayana* plants had increased antioxidant activity and decreased growth and efficiency of photosystem II under salt stress. However, AMF alleviated the negative effects of salt on these plants. Males inoculated with AMF showed improved growth and development parameters, physiological functions, and antioxidant activities under salt stress than that of females (Wu et al., 2016). Thus, salinity has a similar effect on *Populus* to drought, with males being more adaptive to salt stress than females, with a higher growth rate, photosynthetic capacity, and antioxidant activity.

## Metal-Associated Stresses

Although appropriate concentrations of some metals are essential for plants, high concentrations of heavy metals inhibit their growth and development. The effects of lead (Pb) on *P. cathayana* plants were studied. Pb treatment negatively affected both sexes; however, males showed higher plasticity in their photosynthetic activity than females. Moreover, drought increased the sensitivity to Pb, especially in females (Han et al., 2013a). It was shown that AMF increased the Pb uptake and accumulation in the roots of female *P. cathayana* plants, but not male plants (Chen L. et al., 2015). *P. deltoides* females were found to be more susceptible to cadmium (Cd) stress than males, and showed leaf symptoms, lipid peroxidation, and altered cellular ultrastructures. Inoculation with *R. intraradices* decreased the toxic effect of Cd in females via an increase in antioxidant activity and limiting Cd transfer to shoots. However, such effects were not detected in males inoculated with AMF (Chen L. et al., 2016). Different responses of *P. cathayana* males and females in single-sex cultivation or sexual competition under Cd stress were also demonstrated. Females exposed to Cd stress had more serious damage and higher Cd accumulation under intrasexual than intersexual competition. On the contrary, males were less affected under intrasexual competition (Chen J. et al., 2016). *P. cathayana* males showed higher antioxidant activity and chlorophyll contents and were more resistant to aluminum (Al) stress than females (Li et al., 2012). *P. cathayana* males were also more tolerant to copper (Cu) stress because they accumulated higher amounts of Cu in leaves than females (Chen et al., 2013). *P. yunnanensis* female plants had higher ROS levels and less effective protection against high zinc (Zn) concentrations than males (Jiang et al., 2013). *P. cathayana* females were more sensitive to iron (Fe) deficiency and had higher growth inhibition and more serious damage of photosynthesis system II than males (Zhang et al., 2016a). Thus, *Populus* female plants were more sensitive to all the examined metal-associated stresses than the male plants.

## Nutrients

Plant growth is often inhibited by nutrient deficiency. *P. cathayana* males had higher antioxidant activities and less damage of photosystem II under nitrogen (N) and phosphorus (P) deficiency. (Zhang et al., 2014). *P. cathayana* male and female plants showed different responses to N and P deficiency at the proteome level. Most of the changes observed were for proteins involved in the stress response and gene expression regulation, with alterations being greater in females than in males. It was suggested that compared to males, *P. cathayana* females were more sensitive to N and P deficiency and had a more rapid metabolic response (Zhang et al., 2016b). Under limited N and P concentrations, *P. tremula* females produced more flavonoids and condensed tannins and invested more in mineral nutrient acquisition, whereas males had higher growth rates (Randriamanana et al., 2014). The growth of *P. cathayana* females was stimulated more significantly than that of males in intersexual competition under high N concentrations. However, males were more adaptive to low N concentrations in intersexual competition (Chen J. et al., 2015). The effect of potassium (K) deficiency on *P. cathayana* was also investigated; it was observed that females had a significantly lower K content in leaves and stems as well as increased sucrose content than that of males. Therefore, males were less sensitive to K deficiency (Yang et al., 2015). Plant growth is linked to soil nutrients and atmospheric CO_2_, which is necessary for photosynthesis. *P. cathayana* photosynthetic capacities and growth were stimulated by elevated CO_2_ levels, and males had greater biomass production than females (Zhao et al., 2012). Besides, the effects of N supply and elevated CO_2_ levels on *P. cathayana* plants were investigated. Both N deposition and elevated CO_2_ levels individually increased the leaf mass and photosynthetic rate in both sexes; however, the effects were weaker when these conditions were present simultaneously_._ Moreover, males were more adaptive to the combined conditions imposed by elevated CO_2_ levels and N deposition than females (Zhao et al., 2011).

## Other Stresses

In addition to the stresses described above, the effect of other unfavorable environmental conditions on male and female *Populus* individuals was also investigated. Enhanced UV-B radiation affects plants negatively. In *P. cathayana* males, the antioxidant enzymes were more efficient and exhibited greater tolerance to the stress induced by UV-B radiation than that in females (Xu et al., 2010). Sex-related transcriptional alterations were observed in *P. cathayana* under UV-B radiation stress. Genes involved in amino acid metabolism were upregulated in males and downregulated in females. The gene regulation strategy was more effective in males than in females (Jiang et al., 2015). Skewed male:female ratios (male-biased) of *P. purdomii* were observed at high altitudes, where multifactor stress including high UV-B radiation levels is present (Lei et al., 2017). Sex-specific differences at the proteome level were noted in *P. cathayana* under conditions of high UV-B radiation. Alterations in the expression levels were higher in males than in females for proteins, which are mainly involved in translation/transcription/post-transcriptional modification, stress responses, and amino acid metabolism (Zhang et al., 2017). However, male buds of *P. tremula* were more sensitive to UV-B than female buds. Moreover, increased temperature caused more delay in bud maturation in males than in females (Stromme et al., 2015). Female floral buds of *P. tomentosa* were observed to be better adapted to heat and chilling stress than male buds. Temperature treatment resulted in increased activities of catalase, peroxidase, and superoxide dismutase in female floral buds, whereas malondialdehyde content was significantly increased in males (Song et al., 2014). In contrast to buds, *P. cathayana* female plants showed less tolerance to chilling stress than males. Disintegrated chloroplasts and numerous tilted grana stacks were identified in female plants under chilling stress, whereas a higher chlorophyll content and antioxidant activity was observed in males (Zhang et al., 2011). *P. cathayana* males had more effective protection against chilling stress at the proteome level than the females (Zhang et al., 2012). However, for *P. trichocarpa* no significant sex-specific differences in response to low and high temperatures were identified: the time from planting of exposed to stress cuttings to bud break and leaf flush was equal for genders (McKown et al., 2017). Apart from chilling, flooding is another winter stress for *Populus* trees. *P. deltoides* males were found to be more tolerant to winter flooding and demonstrated less oxidative damage than females (Miao et al., 2017). In riparian woodland, the skewed male:female ratio (2:1) of a studied population of *P. angustifolia* was observed. Male trees had higher normalized differences in vegetation indexes than females, whereas other leaf reflectance and photosynthetic gas exchange characteristics were similar in both sexes (Letts et al., 2008). Although, the sex-specific response of *Populus* to abiotic stresses has been primarily studied, some data on the differential sensitivity of males and females to biotic stresses were also reported. Leaf rust disease, which is caused by the fungus, *Melampsora laricis-populina*, was more severe in female *P. cathayana* than in males. *M. laricis-populina* infection resulted in higher antioxidant activities, and less negative effects in males than females (Zhang et al., 2010b). Male and female trees of *P. tremuloides* had similar levels of phenolic glycosides and condensed tannins; however, the correlation of growth rate with these defense chemicals was revealed only in females. It was suggested that males are less sensitive to herbivores and need less phenolic glycosides for defense than females (Stevensa and Esserb, 2009).

## Lack of Sexual Dimorphism for Non-Reproductive Features

Diverse stress responses in male and female *Populus* individuals were observed at genomic, epigenomic, proteomic, and physiological levels in most of the above-mentioned studies. However, recently, no differences between *Populus* sexes were revealed for non-reproductive features. *P. tremula* male and female trees from Umea and Swedish Aspen collections, which were wild-growing and not subjected to a particular experimental stress, were compared regarding morphological and biochemical characteristics, arthropod abundance, sex bias, and transcription profiles. No sexual dimorphism was found, with the exception of the expression of two genes (Potri.019G047300 and Potri.014G155300). The first gene is located in the sex-determining region (Robinson et al., 2014) and has deletion in females (Pakull et al., 2015). Sampling of about 1,300 individuals of *P. trichocarpa* and *P. balsamifera* was tested for identification of sex-specific differences in natural conditions. Seventy functional traits and 26 wood-related traits were assessed and no significant differences between genders for non-reproductive features were revealed. Gender neutrality for non-reproductive traits was suggested in both *P. trichocarpa* and *P. balsamifera* (McKown et al., 2017). Divergence in the results of sex-specific features in *Populus* could be associated with the use of diverse plant material (cuttings, seedlings, or mature trees), conditions (natural or controlled stress), as well as sampling size and composition. Besides, in distinct populations of *Populus* species, physiology and morphology are varied, and stress responses could be associated with a particular species and genotype (Rae et al., 2007).

## Genetic Determination of Sex in *Populus*

*Populus* has an XY sex-determining system, and males and females differ at the genome level by 650 SNPs, which are significantly associated with sex. The sex-associated region in *Populus* is relatively small—about 100 kb (Geraldes et al., 2015). It contains a gene encoding METHYLTRANSFERASE1 (MET1), which is involved in DNA methylation – an important mechanism of plant adaptation to stress (Ashapkin et al., 2016). Gender-specific methylation of the *PbRR9* gene, which is also located in the sex-associated region and encodes cytokinin signaling orthologs of ARABIDOPSIS RESPONSE REGULATOR 16 and 17 (ARR 16 and 17), was revealed in xylem tissues of *P. balsamifera* (Brautigam et al., 2017). Thus, genetic differences between males and females influence not only reproductive organs, but also secondary sex characteristics, and therefore, could lead to gender differences in non-reproductive features.

Further studies will show what role in response to unfavorable environments belongs to gender differences in *Populus*, and data received to date have been summarized in **Table 1**.

**Table 1:** *Populus* sex-specific response to environments.

Stress	Species	Sex-specific response
Drought	*Populus cathayana P. yunnanensis*	Females were more sensitive: had more pronounced decrease of growth, physiological functions, hormone biosynthesis, and photosynthesis, but higher accumulation of reactive oxygen species (ROS).
Salinity	*P. cathayana P. deltoides P. yunnanensis*	Females were more sensitive: had less capacity to restrict Na+ transport from roots to shoots, more reduction of growth and photosynthesis, greater damage of cell organelles, but higher accumulation of ROS.
Metals	*P. cathayana P. deltoides P. yunnanensis*	Females were more sensitive to Pb, Cd, Al, Cu, Zn stress and Fe deficiency: had higher growth inhibition, more damage of photosynthesis system and less antioxidant activity.
Nutrients	*P. cathayana P. tremula*	Females were more sensitive to nutrient deficiency: had higher growth inhibition. Males had greater biomass production under elevated CO_2_ conditions.
UV-B	*P. cathayana P. purdomii P. tremula*	Female trees were more sensitive to UV-B: had less antioxidant activity and amino acid metabolism. However, female buds had less damage under UV-B than male buds.
Temperature	*P. tremula P. tomentosa P. cathayana P. trichocarpa*	Females were more sensitive to chilling stress: had more damage of photosynthesis system and less antioxidant activity. However, female buds had increased antioxidant activity and less damage under heat and chilling stress. In *P. trichocarpa*, no sex-specific response to temperature stress was revealed.

## Conclusion

Considerable information on gender differences of *Populus* species, especially under unfavorable conditions, has been accumulating. Most research has shown that male *Populus* plants tend to be more tolerant to environmental stresses, such as drought, salinity, heavy metals, and nutrient deficiency, than female plants. Under unfavorable conditions, males exhibit less reduction in growth rate and photosynthetic activity, as well as less cellular damage and higher antioxidant activities than females. The inverse situation was observed for floral buds – females had less damage than males under temperature stress and high UV-B radiation. Thus, it can be suggested that under unfavorable conditions, the strategy of male trees is to maintain their growth and vegetative mass, whereas females aim to preserve their reproductive organs. However, some recent studies showed no differences between *Populus* sexes for non-reproductive traits and adaptation strategies. Controversial results could be associated with genotype differences, definite stress conditions, types of plant material, sampling sizes, etc. Understanding sex-specific differences is crucial for basic research and effective practical applications in dioecious species. Further studies are needed to conclude whether there is a difference in secondary sex characteristics between *Populus* sexes.

## Author Contributions

NM, EB, AS, AK, and AD wrote the manuscript. All the authors revised the work critically for important intellectual content, approved the version to be published, and agreed to be accountable for all aspects of the work in ensuring that questions related to the accuracy or integrity of any part of the work are appropriately investigated and resolved.

## Conflict of Interest Statement

The authors declare that the research was conducted in the absence of any commercial or financial relationships that could be construed as a potential conflict of interest.

## Footnotes

1 https://phytozome.jgi.doe.gov/pz/portal.html

2 http://popgenie.org/

## References

[B1] Ashapkin V. V. Kutueva L. I. Vanyushin B. F. (2016). Plant DNA methyltransferase genes: multiplicity, expression, methylation patterns. *Biochemistry* 81 141–151. 10.1134/S0006297916020085 27260394

[B2] Brautigam K. Soolanayakanahally R. Champigny M. Mansfield S. Douglas C. Campbell M. M. (2017). Sexual epigenetics: gender-specific methylation of a gene in the sex determining region of *Populus balsamifera*. *Sci. Rep.* 7:45388. 10.1038/srep45388 28345647 PMC5366940

[B3] Chen F. Chen L. Zhao H. Korpelainen H. Li C. (2010). Sex-specific responses and tolerances of *Populus cathayana* to salinity. *Physiol. Plant.* 140 163–173. 10.1111/j.1399-3054.2010.01393.x 20561244

[B4] Chen F. Zhang S. Jiang H. Ma W. Korpelainen H. Li C. (2011). Comparative proteomics analysis of salt response reveals sex-related photosynthetic inhibition by salinity in *Populus cathayana* cuttings. *J. Proteome Res.* 10 3944–3958. 10.1021/pr200535r 21761936

[B5] Chen J. Dong T. Duan B. Korpelainen H. Niinemets U. Li C. (2015). Sexual competition and N supply interactively affect the dimorphism and competiveness of opposite sexes in *Populus cathayana*. *Plant Cell Environ.* 38 1285–1298. 10.1111/pce.12477 25366665

[B6] Chen J. Duan B. Xu G. Korpelainen H. Niinemets U. Li C. (2016). Sexual competition affects biomass partitioning, carbon-nutrient balance, Cd allocation and ultrastructure of *Populus cathayana* females and males exposed to Cd stress. *Tree Physiol.* 36 1353–1368. 10.1093/treephys/tpw054 27344063

[B7] Chen L. Hu X. Yang W. Xu Z. Zhang D. Gao S. (2015). The effects of arbuscular mycorrhizal fungi on sex-specific responses to Pb pollution in *Populus cathayana*. *Ecotoxicol. Environ. Saf.* 113 460–468. 10.1016/j.ecoenv.2014.12.033 25553418

[B8] Chen L. Wang L. Chen F. Korpelainen H. Li C. (2013). The effects of exogenous putrescine on sex-specific responses of *Populus cathayana* to copper stress. *Ecotoxicol. Environ. Saf.* 97 94–102. 10.1016/j.ecoenv.2013.07.009 23938041

[B9] Chen L. Zhang D. Yang W. Liu Y. Zhang L. Gao S. (2016). Sex-specific responses of *Populus deltoides* to *Glomus intraradices* colonization and Cd pollution. *Chemosphere* 155 196–206. 10.1016/j.chemosphere.2016.04.049 27115844

[B10] Chen L. Zhang S. Zhao H. Korpelainen H. Li C. (2010). Sex-related adaptive responses to interaction of drought and salinity in *Populus yunnanensis*. *Plant Cell Environ.* 33 1767–1778. 10.1111/j.1365-3040.2010.02182.x 20545878

[B11] Eckenwalder J. E. (1996). “Systematics and evolution of *Populus*,” in *Biology of Populus and Its Implications for Management and Conservation. Part I*, eds Stettler R. F. Bradshaw H. D. Jr. Heilman P. E. Hinckley T. M. (ottawa, ON: NRC Research Press), 7–32.

[B12] Ellis B. Jansson S. Strauss S. Tuskan G. A. (2010). “Why and how *Populus* became a “model tree”,” in *Genetics and Genomics of Populus*, eds Sansson S. Bhalerao R. Groover A. (New York, NY: Springer Science+Business Media), 3–14.

[B13] Geraldes A. Hefer C. A. Capron A. Kolosova N. Martinez-Nunez F. Soolanayakanahally R. Y. (2015). Recent Y chromosome divergence despite ancient origin of dioecy in poplars (*Populus*). *Mol. Ecol.* 24 3243–3256. 10.1111/mec.13126 25728270

[B14] Han Y. Wang L. Zhang X. Korpelainen H. Li C. (2013a). Sexual differences in photosynthetic activity, ultrastructure and phytoremediation potential of *Populus cathayana* exposed to lead and drought. *Tree Physiol.* 33 1043–1060. 10.1093/treephys/tpt086 24186942

[B15] Han Y. Wang Y. Jiang H. Wang M. Korpelainen H. Li C. (2013b). Reciprocal grafting separates the roles of the root and shoot in sex-related drought responses in *Populus cathayana* males and females. *Plant Cell Environ.* 36 356–364. 10.1111/j.1365-3040.2012.02578.x 22788254

[B16] Harfouche A. Meilan R. Altman A. (2014). Molecular and physiological responses to abiotic stress in forest trees and their relevance to tree improvement. *Tree Physiol.* 34 1181–1198. 10.1093/treephys/tpu012 24695726

[B17] Jiang H. Korpelainen H. Li C. (2013). *Populus yunnanensis* males adopt more efficient protective strategies than females to cope with excess zinc and acid rain. *Chemosphere* 91 1213–1220. 10.1016/j.chemosphere.2013.01.041 23415309

[B18] Jiang H. Peng S. Zhang S. Li X. Korpelainen H. Li C. (2012). Transcriptional profiling analysis in *Populus yunnanensis* provides insights into molecular mechanisms of sexual differences in salinity tolerance. *J. Exp. Bot.* 63 3709–3726. 10.1093/jxb/ers064 22442418 PMC3388841

[B19] Jiang H. Zhang S. Feng L. Korpelainen H. Li C. (2015). Transcriptional profiling in dioecious plant *Populus cathayana* reveals potential and sex-related molecular adaptations to solar UV-B radiation. *Physiol. Plant.* 153 105–118. 10.1111/ppl.12224 24813713

[B20] Lei Y. Chen K. Jiang H. Yu L. Duan B. (2017). Contrasting responses in the growth and energy utilization properties of sympatric *Populus* and *Salix* to different altitudes: implications for sexual dimorphism in Salicaceae. *Physiol. Plant.* 159 30–41. 10.1111/ppl.12479 27300648

[B21] Letts M. G. Phelan C. A. Johnson D. R. Rood S. B. (2008). Seasonal photosynthetic gas exchange and leaf reflectance characteristics of male and female cottonwoods in a riparian woodland. *Tree Physiol.* 28 1037–1048. 18450568 10.1093/treephys/28.7.1037

[B22] Li J. Y. Xu X. Yang P. Wang B. X. Wang Z. F. Li X. F. (2012). [Effects of aluminum stress on ecophysiological characteristics of male and female *Populus cathayana* seedlings]. *Ying Yong Sheng Tai Xue Bao* 23 45–50. 22489478

[B23] Li L. Zhang Y. Luo J. Korpelainen H. Li C. (2013). Sex-specific responses of *Populus yunnanensis* exposed to elevated CO2 and salinity. *Physiol. Plant.* 147 477–488. 10.1111/j.1399-3054.2012.01676.x 22897484

[B24] Li Y. Duan B. Chen J. Korpelainen H. Niinemets U. Li C. (2016). Males exhibit competitive advantages over females of *Populus deltoides* under salinity stress. *Tree Physiol.* 36 1573–1584. 10.1093/treephys/tpw070 27587482

[B25] Li Z. Wu N. Liu T. Chen H. Tang M. (2015a). Effect of arbuscular mycorrhizal inoculation on water status and photosynthesis of *Populus cathayana* males and females under water stress. *Physiol. Plant* 10.1111/ppl.12336 [Epub ahead of print]. 25720810

[B26] Li Z. Wu N. Liu T. Chen H. Tang M. (2015b). Sex-related responses of *Populus cathayana* shoots and roots to AM fungi and drought stress. *PLOS ONE* 10:e0128841. 10.1371/journal.pone.0128841 26102587 PMC4478039

[B27] McKown A. D. Klapste J. Guy R. D. Soolanayakanahally R. Y. La Mantia J. Porth I. (2017). Sexual homomorphism in dioecious trees: extensive tests fail to detect sexual dimorphism in Populus †. *Sci. Rep.* 7:1831. 10.1038/s41598-017-01893-z 28500332 PMC5431824

[B28] Miao L. F. Yang F. Han C. Y. Pu Y. J. Ding Y. Zhang L. J. (2017). Sex-specific responses to winter flooding, spring waterlogging and post-flooding recovery in *Populus deltoides*. *Sci. Rep.* 7:2534. 10.1038/s41598-017-02765-2 28566759 PMC5451430

[B29] Pakull B. Kersten B. Luneburg J. Fladung M. (2015). A simple PCR-based marker to determine sex in aspen. *Plant Biol.* 17 256–261. 10.1111/plb.12217 24943351

[B30] Peng S. Jiang H. Zhang S. Chen L. Li X. Korpelainen H. (2012). Transcriptional profiling reveals sexual differences of the leaf transcriptomes in response to drought stress in *Populus yunnanensis*. *Tree Physiol.* 32 1541–1555. 10.1093/treephys/tps110 23148036

[B31] Rae A. M. Street N. R. Rodríguez-Acosta M. (2007). “Populus trees,” in *Forest Trees*, ed. Kole C. (Berlin: Springer Berlin Heidelberg), 1–28.

[B32] Randriamanana T. R. Nybakken L. Lavola A. Aphalo P. J. Nissinen K. Julkunen-Tiitto R. (2014). Sex-related differences in growth and carbon allocation to defence in *Populus tremula* as explained by current plant defence theories. *Tree Physiol.* 34 471–487. 10.1093/treephys/tpu034 24852570

[B33] Robinson K. M. Delhomme N. Mahler N. Schiffthaler B. Onskog J. Albrectsen B. R. (2014). *Populus tremula* (European aspen) shows no evidence of sexual dimorphism. *BMC Plant Biol.* 14:276. 10.1186/s12870-014-0276-5 25318822 PMC4203875

[B34] Song Y. Ma K. Ci D. Zhang Z. Zhang D. (2014). Biochemical, physiological and gene expression analysis reveals sex-specific differences in *Populus tomentosa* floral development. *Physiol. Plant.* 150 18–31. 10.1111/ppl.12078 23773142

[B35] Stevensa M. T. Esserb S. M. (2009). Growth–defense tradeoffs differ by gender in dioecious trembling aspen (*Populus tremuloides*). *Biochem. Syst. Ecol.* 37 567–573.

[B36] Stromme C. B. Julkunen-Tiitto R. Krishna U. Lavola A. Olsen J. E. Nybakken L. (2015). UV-B and temperature enhancement affect spring and autumn phenology in *Populus tremula*. *Plant Cell Environ.* 38 867–877. 10.1111/pce.12338 24689776

[B37] Tuskan G. A. Difazio S. Jansson S. Bohlmann J. Grigoriev I. Hellsten U. (2006). The genome of black cottonwood, *Populus trichocarpa* (Torr. & Gray). *Science* 313 1596–1604. 10.1126/science.1128691 16973872

[B38] Wu N. Li Z. Wu F. Tang M. (2016). Comparative photochemistry activity and antioxidant responses in male and female *Populus cathayana* cuttings inoculated with arbuscular mycorrhizal fungi under salt. *Sci. Rep.* 6:37663. 10.1038/srep37663 27898056 PMC5127190

[B39] Xu X. Peng G. Wu C. Korpelainen H. Li C. (2008a). Drought inhibits photosynthetic capacity more in females than in males of *Populus cathayana*. *Tree Physiol.* 28 1751–1759. 18765380 10.1093/treephys/28.11.1751

[B40] Xu X. Yang F. Xiao X. Zhang S. Korpelainen H. Li C. (2008b). Sex-specific responses of *Populus cathayana* to drought and elevated temperatures. *Plant Cell Environ.* 31 850–860. 10.1111/j.1365-3040.2008.01799.x 18284585

[B41] Xu X. Zhao H. Zhang X. Hanninen H. Korpelainen H. Li C. (2010). Different growth sensitivity to enhanced UV-B radiation between male and female *Populus cathayana*. *Tree Physiol.* 30 1489–1498. 10.1093/treephys/tpq094 21071771

[B42] Yang Y. Jiang H. Wang M. Korpelainen H. Li C. (2015). Male poplars have a stronger ability to balance growth and carbohydrate accumulation than do females in response to a short-term potassium deficiency. *Physiol. Plant.* 155 400–413. 10.1111/ppl.12325 25615581

[B43] Zhang S. Chen F. Peng S. Ma W. Korpelainen H. Li C. (2010a). Comparative physiological, ultrastructural and proteomic analyses reveal sexual differences in the responses of *Populus cathayana* under drought stress. *Proteomics* 10 2661–2677. 10.1002/pmic.200900650 20455211

[B44] Zhang S. Feng L. Jiang H. Ma W. Korpelainen H. Li C. (2012). Biochemical and proteomic analyses reveal that *Populus cathayana* males and females have different metabolic activities under chilling stress. *J. Proteome Res.* 11 5815–5826. 10.1021/pr3005953 23072643

[B45] Zhang S. Jiang H. Peng S. Korpelainen H. Li C. (2011). Sex-related differences in morphological, physiological, and ultrastructural responses of *Populus cathayana* to chilling. *J. Exp. Bot.* 62 675–686. 10.1093/jxb/erq306 20926551 PMC3003813

[B46] Zhang S. Jiang H. Zhao H. Korpelainen H. Li C. (2014). Sexually different physiological responses of *Populus cathayana* to nitrogen and phosphorus deficiencies. *Tree Physiol.* 34 343–354. 10.1093/treephys/tpu025 24739232

[B47] Zhang S. Lu S. Xu X. Korpelainen H. Li C. (2010b). Changes in antioxidant enzyme activities and isozyme profiles in leaves of male and female *Populus cathayana* infected with *Melampsora larici-populina*. *Tree Physiol.* 30 116–128. 10.1093/treephys/tpp094 19917640

[B48] Zhang S. Zhang Y. Cao Y. Lei Y. Jiang H. (2016a). Quantitative proteomic analysis reveals *Populus cathayana* females are more sensitive and respond more sophisticatedly to iron deficiency than males. *J. Proteome Res.* 15 840–850. 10.1021/acs.jproteome.5b00750 26842668

[B49] Zhang S. Zhou R. Zhao H. Korpelainen H. Li C. (2016b). iTRAQ-based quantitative proteomic analysis gives insight into sexually different metabolic processes of poplars under nitrogen and phosphorus deficiencies. *Proteomics* 16 614–628. 10.1002/pmic.201500197 26698923

[B50] Zhang Y. Feng L. Jiang H. Zhang Y. Zhang S. (2017). Different proteome profiles between male and female *Populus cathayana* exposed to UV-B radiation. *Front. Plant Sci.* 8:320. 10.3389/fpls.2017.00320 28326097 PMC5339244

[B51] Zhao H. Li Y. Zhang X. Korpelainen H. Li C. (2012). Sex-related and stage-dependent source-to-sink transition in *Populus cathayana* grown at elevated CO(2) and elevated temperature. *Tree Physiol.* 32 1325–1338. 10.1093/treephys/tps074 22918961

[B52] Zhao H. Xu X. Zhang Y. Korpelainen H. Li C. (2011). Nitrogen deposition limits photosynthetic response to elevated CO2 differentially in a dioecious species. *Oecologia* 165 41–54. 10.1007/s00442-010-1763-5 20809407

